# GLANet: global and local anomaly network for distributed cyber threat detection using FL

**DOI:** 10.1038/s41598-026-58602-y

**Published:** 2026-06-19

**Authors:** Asia Othman Aljahdali

**Affiliations:** Cybersecurity Department, College of Computer Sciences and Engineering, University of Jeddah, Jeddah, Saudi Arabia

**Keywords:** FL, Anomaly detection, Privacy preservation, Differential privacy, And IoT security, And cyber threat detection, Engineering, Mathematics and computing

## Abstract

This paper introduces GLANet, a novel FL-based framework designed for real-time, privacy-preserving anomaly detection in distributed network environments. GLANet is a FL-based framework that integrates a lightweight convolutional neural network (CNN) as a local anomaly detector at each distributed node with a global model aggregation mechanism based on federated averaging (FedAvg). The framework incorporates consistency regularization to align local and global model parameters, ensuring that node-specific threat patterns are preserved while enabling network-wide generalization. Differential privacy is employed through the injection of calibrated Gaussian noise into model updates before transmission to the central server, providing formal privacy guarantees without compromising detection performance. The experimental findings reveal that GLANet achieves a high detection accuracy of 97.8% on the CICIDS 2017 dataset, surpassing traditional FL baselines that achieved 92.7%. The model also achieves superior precision (96.5%), recall (97.0%), and F1 Score (96.8%), reflecting a well-balanced anomaly detection performance. Additionally, GLANet reduces communication costs by 37.5% and achieves a lower privacy loss of ε = 0.8 compared to 1.5 in baseline methods. These findings demonstrate the potential of GLANet as an effective, scalable, and secure anomaly detection framework for comprehensive defense against evolving cyber threats in dynamic and distributed network environments.

## Introduction

The rapid proliferation of cyber threats in distributed network environments has heightened the need for advanced security solutions capable of adapting to evolving attack patterns without compromising data privacy. The latest reports reveal that cybersecurity attacks keep growing in terms of occurrence and complexity, distributed networks and IoT ecosystems are the main target of malicious actors^1^. Conventional centralized cybersecurity frameworks fail to adequately capture the localized and heterogeneous attack patterns arising from diverse distributed nodes, and thus new distributed models are needed and can work efficiently in resource-restricted contexts without compromising security assurances.

The issue of privacy has become a central obstacle to cooperative cybersecurity models and specifically in the multi organizational and multi domain contexts. As Liu et al.^2^ proved, although blockchain-enhanced FL solutions offer high security basis, they have severe scalability challenges when implemented into large distributed network. Federated learning (FL) is an alternative approach to the fundamental privacy barriers that allows raw data to be trained on multiple nodes without sharing it, thus mitigating privacy threats and enabling nodes to collaboratively detect threats^3^. Nevertheless, more recent studies have shown that the standard FL frameworks do not pay much attention to the presence of attacks that are specific to the node, which makes the need to have frameworks that integrate the local anomaly detection feature with the worldwide threat recognition imperative.

This paper presents the GLANet, an emerging FL-based framework, which incorporates local and global anomaly detection modules to overcome the constraints identified in the recent literature. The suggested solution is based on the experience of Zhang et al.^4^ who noted that structural relationships should be captured in network data and the scalability and resource efficiency limitations of network data illustrated by Hassan et al.^1^ should be tackled. Compared with the current methods that optimize only local or global, GLANet is a holistic solution that improves the accuracy in detection and the communication efficiency at the same time based on the innovative design of hybrid architecture.

With the cyber threats becoming more pronounced and the distributed networks growing, the weaknesses of the traditional centralized systems of cybersecurity become more evident. Recent detailed examinations have demonstrated that the current FL solutions to anomaly detection are challenged with major issues on privacy protection, communication expenses, and inter-domain generalization^5^. The motivation for this study stems from the critical need for an FL-based framework that guarantees strong privacy protection and achieves high detection accuracy across diverse attack scenarios, and is more efficient in communication in a heterogeneous network structure and addresses the deployment complexity challenge that is observed in the existing state-of-the-art solutions.

The underlying problem that this study seeks to answer is the fact that the existing FL designs are not capable of adequately trading off various competing goals in distributed cybersecurity systems. Recent research has established that the currently available solutions have severe shortcomings: blockchain-related solutions are limited in scalability^2^, privacy preserving algorithms lack theoretical protection against sophisticated attacks^5^, and graph-based solutions tend to have significant overhead issues^4^. These shortcomings underscore the need for a unified framework capable of simultaneously supporting local threat detection, global pattern recognition, privacy preservation, and efficient communication without compromising practical deployability. Nevertheless, despite the remarkable progress in FL applied to cybersecurity, the existing methods have inherent weaknesses that make them impossible to implement in the distributed network setting. Liu et al.^2^ found that blockchain-enhanced FL systems were limited in scalability to critical extent especially in large scale IoT deployment where computation 2 overheads are prohibitive. Alhindi et al.^5^ found that there is a gap existing in the privacy-preserving mechanisms, as they were not developed with adequate theoretical foundations to resist advanced adversarial attacks and thus provide vulnerability in practical implementation situations. Zhang et al.^4^ have shown that the existing graph-based methods have high memory and cannot adapt to dynamic network structures, which inhibits their use in constrained resource settings. The resource intensity problems of multi-layered security frameworks were mentioned by Hassan et al.^1^, giving rise to the basic trade-offs between security comprehensiveness and feasible deployability. Moreover, the authors Hendaoui et al.^3^ highlighted important problems that have to do with dataset bias and limited cross domain generalization that limits the usefulness of FL systems in the context of different organizational setups. All these restrictions testify to the dire necessity of a single framework capable of simultaneously attaining local anomaly detection, global threat recognition, preservation of privacy, and efficiency of communication and remaining viable to deployment in heterogeneous distributed networks.

The key contributions of our study are:

- *GLANet framework development* Presentation of a new FL framework that has the ability to integrate local and global detection of anomalies into one system, solving the integration problems that have been observed in the recent extensive literature.
- *Enhanced detection performance* It has been shown that localized threat representation and models of the world work together to significantly improve the detection performance, and that can be performed better than the current 2025 state-of-the-art methods.
- *Formal privacy guarantees via differential privacy* GLANet integrates differential privacy into the FL pipeline by introducing calibrated Gaussian noise into local model updates prior to global aggregation. This mechanism provides a rigorous ε-differential privacy guarantee, formally bounding information leakage and offering stronger theoretical protection against adversarial inference attacks compared to conventional FL approaches that rely solely on data locality for privacy protection.
- *Optimization of communication efficiency* Innovative development of strategies that incur significant cost and communication overhead reductions, overcoming the scalability constraints of the new blockchain-enhanced and graph-based systems.
- *Comprehensive experimental validation* Extensive experiments demonstrate that GLANet achieves superior detection rates, reduced communication overhead, and stronger privacy preservation compared to state-of-the-art FL models.

The paper is divided into various sections that are of paramount importance in covering the proposed GLANet framework. The introduction provides the research background and rationale on the basis of recent advances in FL cybersecurity. The literature review focuses on the analysis of related works with a focus on the latest developments and shows significant research gaps. The methodology section elaborates the design, implementation and the theoretical basis of GLANet. The experimental findings show that GLANet can be used to achieve better detection rates and minimize communication efficiency and privacy protection. Lastly, the conclusion summarizes the main findings and plans of further research aiming at developing FL-based cybersecurity solutions.

What fundamentally distinguishes GLANet from existing FL-based anomaly detection frameworks is its simultaneous and jointly optimized integration of local anomaly detection with global threat recognition through a unified objective function. Existing FL-based IDS frameworks predominantly operate in one of two modes: either they train a single global model with no node-specific adaptation, losing sensitivity to local attack patterns, or they apply purely local detection without any global aggregation, sacrificing generalization across the network. GLANet bridges this dichotomy through three mechanisms not found together in prior work: (1) a per-node lightweight CNN that learns local node-specific anomaly signatures from heterogeneous traffic distributions without sharing raw data; (2) a FedAvg-based global aggregation that synthesizes network-wide threat knowledge from all participating nodes; and (3) a consistency regularization term that explicitly penalizes divergence between local and global model parameters, enforcing a controlled trade-off between local specificity and global generalization. This three-component design allows GLANet to detect both common attack patterns that manifest across multiple nodes and rare, node-specific intrusions that would be suppressed or diluted in a purely global model.

## Literature review

The recent developments in the field of cybersecurity have indicated the unprecedented significance of anomaly-related detection in protecting distributed systems against advanced attacks. Conventional approaches, including statistical analysis and rule-based systems, have proven insufficient to accommodate the dynamic and evolving nature of cyber threats. These approaches typically suffer from high false positive rates and frequently miss genuine attacks^6^.

FL has become a disruptive method of facilitating joint model training whilst maintaining privacy of data across the distributed nodes. The risks of centralized data collection are effectively mitigated when FL is employed to enable local training on sensitive data, with only aggregated model updates shared across nodes. Such an approach has been shown to achieve competitive anomaly detection accuracy without compromising user privacy^7^. Wang et al.^6^ also suggested the use of an attention-based graph neural network to identify cross-level and cross-department network attacks where it can be proposed to train the models collaboratively without affecting data privacy on distributed devices.

### Privacy-preserving FL

Privacy-preserving integration with FL has become the focus of much cybersecurity research in the recent past. Khalaf et al.^8^ have provided a hybrid differential privacy method to use in sharing of knowledge across IoT platforms and have shown significant gains in the accuracy of the model without violating privacy. A detailed framework of differentiating privacy with FL in IoT settings was introduced by Zhao et al.^9^, which guarantees a high level of privacy protection and also does not decrease the detection rates.

Homomorphic encryption has turned out to be an effective method of protecting FL traffic. A new framework based on homomorphic encryption to detect intrusion privacy in resource-constrained Internet-of-Vehicles networks was proposed by Duc Manh et al.^10^. Their method allows the centralized servers to compute on quantum-secure encrypted ciphertexts bypassing decryption. Likewise, Pan et al.^11^ designed FedSHE, an efficient FL scheme with adaptive segmented CKKS homomorphic encryption, which is better in performance, in terms of model accuracy, computational efficiency, and security level.

### Blockchain-enhanced FL

FL has been adopted in recent times in conjunction with blockchain technology to deal with the issue of trust and security in distributed environments. The study by Liu et al.^2^ offered an extensive literature review on the adaptation of security and decentralized knowledge enhancement of FL with the blockchain technology. They found that the introduction of blockchains provides a high data integrity level and tamper-resistant model updates.

Hassan et al.^12^ proposed a scalable smart city architecture with blockchain and FL solutions to IoT security. Their BFLIoT system showed great enhancements in throughput, reliability, and energy use without compromising a strong security of the system against high-tech cyberattacks. Wu et al.^13^ suggested a hierarchical federated learning architecture that is built on blockchain technology and is particularly aimed at the training of non-IID data without the loss of data privacy or security. Furthermore, blockchain-based frameworks for secure data sharing, such as those using optimized deep belief networks for disease diagnosis, highlight the broad applicability of distributed trust mechanisms in sensitive data environments^14^. Sharma and Shambharkar proposed a hybrid framework integrating cryptographic optimization, Ethereum blockchain, and IPFS-based decentralized storage with an optimized deep belief neural network (ODBNN), achieving 99.25% accuracy on benchmark datasets while ensuring tamper-proof data management. Furthermore, recent advances in lightweight blockchain integration for FL have addressed the computational overhead limitations of earlier blockchain-FL systems. Rezaei et al.^15^ proposed BCH-FedSSL, a lightweight blockchain-based defense method for federated self-supervised learning that decentralizes the model aggregation process to counter model poisoning attacks, specifically randomized weight poisoning attacks (RWPA). By incorporating blockchain technology to verify and exclude malicious model updates, BCH-FedSSL significantly reduces the resource burden of consensus mechanisms while maintaining robust tamper resistance and model integrity assurances, making it more suitable for deployment in resource-constrained distributed network environments.

### Edge computing and resource optimization

The application of FL in edge computing systems has brought with it some special issues concerning resource limitations and communication effectiveness. In a systematic review of FL applications in cloud and edge security, Li et al.^16^ reported that FL results in the 25% privacy risk and 40% threat detection improvement of critical infrastructure.

Heidari et al.^17^ came up with a smart deep FL framework that can be used to improve security in the IoT-enabled edge computing system. Their solution can solve the twofold problems of scarcity of communication resources and power constraints of the device by using creative resources allocation policies. Recently, Yuan et al.^18^ introduced a FL system with the assistance of UAVs and incorporates the use of wireless energy harvesting to address the problem of energy scarcity in distributed learning. Recently, the multi-layered security architecture proposed for IoMT systems by Sharma and Shambharkar^19^, which integrates dynamic key management via Dynamic Adaptive Deep Reinforcement Learning (DA-DRL), AES encryption, IPFS-based decentralized storage, and a dependable intrusion detection framework, represents a complementary approach to resource-aware distributed security in constrained environments.

### Advanced neural architectures

The application of graph neural networks has demonstrated good outcomes in anomaly detection in FL. The paper by Tang et al.^20^ proposed a self-boosted knowledge distillation method of effective federated graph anomaly detection, which showed a higher quality of work in a distributed setting. Kong et al.^21^ designed a federated graph anomaly detection framework based on the contrastive self-supervised learning, which is effective to represent complex relationships in network data without violating privacy.

The review of the advances in the field of artificial intelligence in anomaly detection of telecom networks presented by Chen et al.^22^ displays the opportunities of graph neural networks and FL to overcome the challenge of scalability and privacy. Outlining the relevance of topological relationships in the distributed network settings, Zhang et al.^4^ developed a spatial structure-based graph neural network framework to detect network data anomalies. In the context of IoMT environments, the Multi-attention DeepCRNN framework proposed by Sharma and Shambharkar^23^ demonstrates that combining multi-head attention mechanisms with CNN-RNN hybrid architectures can yield both high detection accuracy and model explainability in medical IoT settings, offering interpretable insights into feature importance and temporal dependencies in network traffic patterns.

### Multi-objective optimization

The recent studies have been oriented towards the solution of multi-objective character of the FL systems, in particular, the performance, the privacy, and the resource efficiency balance. Li et al.^24^ explored the concepts of multi-objective FL mechanisms that compromise global performance with personal fairness, presenting new optimization mechanisms to resolve the trade-offs of distributed learning systems.

Zhang et al.^25^ researched on the topic of fairness-conscious multi-objective optimization in FL and suggested methods where both utility and fairness are the main goals. Their research shows that well thought multi-objective strategies can obtain enhanced system performance without unfair treatment between the involved nodes.

### Comprehensive security analysis

Recent thorough surveys have found the significant challenges and opportunities in FL security. Alhindi et al.^5^ performed a detailed review of split learning with privacy maintained and put forward the fundamental limitations and suggested possible research directions. They indicate that stronger privacy-preserving mechanisms must be developed that can be resistant to advanced adversarial attacks.

El Mestari and Lenzini^26^ offered a methodical review of the subject of data privacy assurance in machine learning models and address the different threats to data safety and offer holistic models of privacy-adhering FL. In the field of FL, Liu et al.^27^ introduced a thorough overview of anomaly detection and defense methods and classified types of attack vectors and defense tools (Table 1).

**Table 1:** Comprehensive comparative analysis of recent FL approaches (2025).

Refs.	Technique	Key innovation	Privacy method	Accuracy (%)	Deployment complexity	Limitations
^2^	Blockchain-FL integration	Decentralized knowledge enhancement with security analysis	Blockchain consensus	94.2	Very high	Scalability concerns in large-scale deployments, high computational overhead
^5^	Privacy preserving split learning	Comprehensive experimental analysis of split learning privacy	Split learning + DP	91.5	Moderate	Limited evaluation on real-world adversarial scenarios, theoretical gaps
^4^	Spatial graph neural networks	Structure-aware anomaly detection with spatial features	Local processing	93.7	High	High memory requirements, complexity in dynamic network topologies
^1^	Edge-IoT security integration	Multi-layered defense with ML and blockchain combination	Multi-layer encryption	92.8	Very high	Resource intensity for IoT devices, integration complexity
^3^	Threat intelligence FL	Real-world dataset generation with cyber threat intelligence	Standard FL	95.1	Low	Dataset bias concerns, limited cross-domain generalization

### Research gap

Despite significant progress in FL-based cybersecurity systems, several research gaps continue to impede the practical implementation of distributed anomaly detection framework. Liu et al.^2^ found that existing blockchain-FL solutions lack scalability and high computational costs, especially in IoT settings with limited resources, and Alhindi et al.^5^ found that existing privacy-preserving solutions lacked adequate theoretical underpinnings to ensure that powerful adversarial attacks are mitigated in practice. Zhang et al.^4^ mentioned the issues of managing dynamic network topology and large memory demands in graph-based methods, and the problem of dataset bias and limited cross-domain generalization in FL systems in detecting threats was critical as pointed out in Hendaoui et al.^3^. Hassan et al.^1^ observed that the main trade-off between security comprehensiveness and practical deployability is that multi-layered security frameworks are very resource-intensive at the edge devices, adding an additional external burden to resource complexity. Overall, the existing literature reveals a critical absence of unified frameworks capable of simultaneously combining local node-specific anomaly detection with global threat recognition while maximizing communication efficiency, privacy protection, and detection accuracy across heterogeneous distributed networks, there is an immediate necessity to come up with innovative schemes that can make joint trade-offs between these competing goals with the help of new design paradigms and multi-objective optimization techniques.

## Methodology

This section introduces the methodology behind the development of the proposed model, GLANet, that combines global and local anomaly detection towards distributed cyber threat detection through FL. The methodology will be splintered into the following sections: dataset collection, data preprocessing, proposed model, training process and evaluation metrics.

### Dataset collection and description

To train and test the suggested GLANet model, I gathered and used three different sets of data, encompassing very diverse types of cyber threats and attack cases. The datasets contain both publicly available benchmarks and simulated data to depict the real-life distributed environments, specifically the heterogeneous network nodes, especially the IoT devices. In this subsection, I elaborate on the nature, features, and applicability of each of the datasets applied in the study.

#### NSL-KDD dataset

The NSL-KDD dataset is a commonly used reference in research on network anomaly detection based on the original KDD Cup 1999 dataset. It overcomes various limitations of the predecessor including elimination of duplicative records which has made it more balanced and reflects real world network traffic.

- *NSL-KDD dataset* is a collection of network economic records which are either classified as normal or as one of the attack types. The attacks are classified into four major classes.
- *Denial of service (DoS)* An effort to disconnect network services by flooding the system with traffic of ill will.
- *Probe attacks* Testing to obtain information regarding the target network, usually a pre-cursor before other more serious attacks.
- *User to root (U2R) attacks* Attacks in which a user is able to access a system illegally and acquire root access.
- *Remote to local (R2L) attacks* An attack where a malicious individual obtains access to a system without the appropriate credentials.

The data has 41 features, which include simple network packet attributes (e.g., duration, protocol type), content attributes (e.g., number of failed logins), and traffic attributes (e.g., number of connections). All records are marked as being normal or of one of the attack types.

#### CICIDS 2017 dataset

Another benchmark database that can be used when analyzing the intrusion detection systems is the CICIDS 2017 dataset, which is indicative of modern network attacks and network traffic. It features contemporary threats that are common in the current network settings, which form a holistic verification platform to anomaly detection models.

The CICIDS 2017 dataset was developed by the Canadian Institute for Cybersecurity and contains approximately 2.8 million labeled network flow records generated over five consecutive days using the ISCX network traffic generator. The dataset includes seven attack categories: (1) Brute Force (FTP-Patator, SSH-Patator); (2) DoS and DDoS attacks (Slowloris, Slowhttptest, Hulk, GoldenEye, Heartbleed); (3) Web Attacks (Brute Force, XSS, SQL Injection); (4) Infiltration attacks; (5) Botnet (ARES); (6) Port Scan; and (7) normal background traffic. The dataset consists of 80 network traffic features encompassing time-related and flow-related attributes such as inter-arrival time of packets, source/destination IP addresses, and TCP flags.

The dataset exhibits significant class imbalance, with normal traffic comprising approximately 83% of all records, while certain attack classes such as Infiltration and Heartbleed constitute less than 0.01% of samples. To mitigate the effect of class imbalance, we applied the synthetic minority oversampling technique (SMOTE) to minority attack classes during training to achieve a more balanced class distribution. Stratified sampling was employed during dataset splitting to preserve the proportional class representation in each subset. The final dataset was partitioned as follows: 70% for training, 15% for validation (used for hyperparameter tuning), and 15% for testing. All preprocessing steps described in “Data preprocessing” section, including median imputation, Min–Max normalization, one-hot encoding of categorical features, and mutual information-based feature selection, were applied uniformly across all three splits using parameters estimated exclusively from the training set to prevent data leakage.

This data set consists of 80 network traffic features, such as time-related, flow-related features such as the inter-arrival time of packets, the source/destination IP addresses, and TCP flags. It offers a great variety of features that can be used in deep learning-based anomaly detection.

#### Simulated IoT network data

Besides the benchmark datasets, I created a simulated IoT network dataset that was created with a network traffic simulator. The dataset was tailored to the specifics of the IoT devices, which can have a limited computational capacity, and that they tend to have a different traffic pattern.

The generated IoT dataset in simulation consists of normal and malicious traffic of various IoT devices that are smart cameras, sensors, and smart home devices. The simulations of attacks are:

- *Botnet attacks* The IoT devices have been compromised to be used orchestrated attacks such as DDoS.
- *Firmware exploits* Exploits of the firmware of IoT devices.
- *Data exfiltration* Unauthorized data extraction of sensitive information in the network.

The data has 50 attributes encompassing device attributes (e.g., device type, firmware version), network flow attributes (e.g., packet size, duration), and attack attributes (e.g., abnormal packet frequency).

#### Data distribution across nodes

In order to simulate a FL environment, I distributed each dataset among various nodes depending on the type of data. For instance:

- Nodes that were used to simulate enterprise conditions were assigned NSL-KDD subsets.
- Nodes that stand as the modern network infrastructures were assigned the data of the CICIDS 2017 dataset.
- Simulated IoT network dataset IoT-specific nodes were provided with the simulated data of the IoT network, which takes into consideration the peculiarities of traffic specific to smart devices.

This distribution introduces data heterogeneity across nodes, enabling GLANet to effectively learn both node-independent and node-specific attack patterns.

The effectiveness and privacy-awareness objectives of GLANet are supported through the combination of benchmark datasets and simulated data, providing a comprehensive and diverse evaluation framework, which enables the comprehensive and varied evaluation framework.

### Data preprocessing

The performance of the proposed GLANet model depends critically on effective data preprocessing, which ensures the quality, consistency, and suitability of input data for anomaly detection across distributed nodes. Here, I will detail the procedures followed to clean, normalize and prepare the datasets, which will be used in training.

#### Data cleaning

The initial step to resolve the problems of inconsistencies, missing values and duplicates in the dataset involved data cleaning. Missing values in numerical features were imputed using the feature median, which is robust to outliers. In the case of categorical features, the missing values in the features have been filled with mode imputation which is a replacement of these values with the most common category.

##### Duplicate removal

Duplicate records were identified and removed through similarity checks on key attributes, including IP address, timestamp, and protocol type, in order to prevent biased learning and ensure dataset integrity.

##### Handling missing values

In the case of numerical characteristics, the missing data were imputed through median imputation:

1 $${\mathrm{x}}_{\mathrm{i}}={\mathrm{median}}\left({\mathrm{x}}_{1},{\mathrm{x}}_{2},\dots ,{\mathrm{x}}_{\mathrm{n}}\right),$$

where ${\mathrm{x}}_{\mathrm{i}}$ is the imputed value for the missing entry, and ${\mathrm{x}}_{1},{\mathrm{x}}_{2},\dots ,{\mathrm{x}}_{\mathrm{n}}$ are the non-missing values of the feature.

For categorical features, mode imputation was used:

2 $${\mathrm{x}}_{\mathrm{i}}={\mathrm{mode}}\left({\mathrm{x}}_{1},{\mathrm{x}}_{2},\dots ,{\mathrm{x}}_{\mathrm{n}}\right).$$

#### Normalization

The numerical features were modified using normalization to equalize them to a similar range, which is also significant to make sure that the model does not prefer the features that have higher values than others. Min–Max Normalization was used to bring the features to [0,1]:

3 $${\mathrm{x}}^{\prime } = \frac{{{\mathrm{x}} - \min \left( {\mathrm{x}} \right)}}{{\max \left( {\mathrm{x}} \right) - \min \left( {\mathrm{x}} \right)}},$$

where x is the original feature value; min(x) and max(x) are the minimum and maximum values of the feature, respectively; x′ is the normalized feature value in the range $\left[0,1\right]$.

Normalization stabilizes the learning process and accelerates convergence of gradient-based optimization algorithms.

#### Encoding categorical features

The categorical variables that included the type of protocol and service name were encoded into a numerical form using One-Hot Encoding. This method of encoding was selected due to the fact that it does not allow the model to presuppose any ordinal relation among the categories.

##### One-hot encoding

Let $\mathrm{C}$ be the number of unique categories for a given feature. One-hot encoding creates $\mathrm{C}$ binary features, where each feature indicates the presence of one category:

4 $${\text{Encoded vector}}=\left[0,0,1,0,\ldots ,0\right],$$

where only the position corresponding to the category is set to 1, and all other positions are set to 0.

#### Feature selection

I have used a combination of mutual information analysis and correlation analysis in order to reduce the size of the data and remove irrelevant features. The process aids in choosing the most informative characteristics but prevents multicollinearity.

##### Mutual information analysis

Mutual information is used to determine how much the features and the target variable are dependent on each other. The information mutually shared by X and Y I(X;Y) is computed as:

5 $${\mathrm{I}}\left( {{\mathrm{X}};{\mathrm{Y}}} \right) = \sum\limits_{{{\mathrm{x}} \in {\mathrm{X}}}} {\sum\limits_{{{\mathrm{y}} \in {\mathrm{Y}}}} {\mathrm{p}} } \left( {{\mathrm{x}},{\mathrm{y}}} \right)\log \left( {\frac{{{\mathrm{p}}\left( {{\mathrm{x}},{\mathrm{y}}} \right)}}{{{\mathrm{p}}\left( {\mathrm{x}} \right){\mathrm{p}}\left( {\mathrm{y}} \right)}}} \right),$$

where p(x,y) is the joint probability distribution of X and Y, and p(x) and p(y) are the marginal probability distributions.

##### Correlation analysis

The Pearson correlation coefficient was applied to identify and remove highly correlated features—of the two features X and Y is given by:

6 $${\uprho}_{\mathrm{X},\mathrm{Y}}=\frac{\sum_{\mathrm{i}=1}^{\mathrm{n}}\left({\mathrm{x}}_{\mathrm{i}}-\overline{\mathrm{x}}\right)\left({\mathrm{y}}_{\mathrm{i}}-\overline{\mathrm{y}}\right)}{\sqrt{\sum_{\mathrm{i}=1}^{\mathrm{n}}{\left({\mathrm{x}}_{\mathrm{i}}-\overline{\mathrm{x}}\right)}^{2}}\sqrt{\sum_{\mathrm{i}=1}^{\mathrm{n}}{\left({\mathrm{y}}_{\mathrm{i}}-\overline{\mathrm{y}}\right)}^{2}}},$$

where $\overline{\mathrm{x}}$ and $\overline{\mathrm{y}}$ are the means of $\mathrm{X}$ and $\mathrm{Y}$, respectively. Features with a correlation coefficient greater than 0.9 were considered redundant and removed.

#### Data splitting

I separated the processed data into training and validation and testing sets through stratified sampling to ensure that the corresponding classes are maintained. The proportionate ratios were as:

- *Training set* rest 70% of the data, which is used in training the model.
- *Validation set* 15% of data to be used during hyper parameter selection and model selection.
- *Testing set* 15% of data that is to be used to test the final model performance.

*Stratified sampling*

Stratified sampling is designed to have the same distribution of classes in the splits. All subsets have equal likelihood of a sample of belonging to a particular class which can be used to prevent biased judgments.

7 $${\mathrm{P}}\left( {{\mathrm{Class}} = {\mathrm{c}}\mid {\mathrm{Training}}} \right) = {\mathrm{P}}\left( {{\mathrm{Class}} = {\mathrm{c}}\mid {\mathrm{Validation}}} \right) = {\mathrm{P}}\left( {{\mathrm{Class}} = {\mathrm{c}}\mid {\mathrm{Testing}}} \right),$$

where $\mathrm{c}$ denotes the class label.

These preprocessing functions help to make sure that the data which is supplied to the GLANet model is clean and normalized and relevant in order to detect anomalies effectively and efficiently.

### Problem formulation: data privacy and communication efficiency

In FL for distributed cybersecurity, ensuring data privacy while minimizing communication costs among $\mathrm{N}$ nodes poses a significant challenge. Each node $\mathrm{i}$ (where $\mathrm{i}\in \{1,2,\dots ,\mathrm{N}\}$) holds a local dataset ${\mathcal{D}}_{\mathrm{i}}$. The goal is to train a model that balances local privacy with reduced inter-node communication.

8 $$\mathop {\min }\limits_{{\{ {\mathrm{w}}_{{\mathrm{i}}} \} _{{{\mathrm{i}} = 1}}^{{\mathrm{N}}} }} \frac{1}{{\mathrm{N}}}\sum\limits_{{{\mathrm{i}} = 1}}^{{\mathrm{N}}} {{\mathcal{L}}_{{\mathrm{i}}} } \left( {{\mathrm{w}}_{{\mathrm{i}}} } \right) + \uplambda \sum\limits_{{{\mathrm{i}} = 1}}^{{\mathrm{N}}} \parallel {\mathrm{w}}_{{\mathrm{i}}} - {\mathrm{w}}\parallel ^{2}$$

where ${\mathrm{w}}_{\mathrm{i}}$ is the local model parameter at node i, ${\mathcal{L}}_{\mathrm{i}}\left({\mathrm{w}}_{\mathrm{i}}\right)$ is the loss function for node i, w is the globally aggregated model, *λ* balances local and global consistency.

## Objective functions

1. Privacy objective:
  9 $$\min\sum_{\mathrm{i}=1}^{\mathrm{N}}\left(\parallel {\mathrm{w}}_{\mathrm{i}}-\mathrm{w}{\parallel }^{2}+{\updelta}_{\mathrm{i}}\left({\mathrm{w}}_{\mathrm{i}}\right)\right)$$
2. Communication efficiency objective:
  10 $$\min\sum_{\mathrm{i}=1}^{\mathrm{N}}{\upkappa}_{\mathrm{i}}\left(\frac{\parallel {\mathrm{w}}_{\mathrm{i}}-{\mathrm{w}}_{\mathrm{i}-1}{\parallel }^{2}}{\mathrm{T}}\right)$$

Notations: ${\updelta}_{\mathrm{i}}\left({\mathrm{w}}_{\mathrm{i}}\right)$: Privacy-preserving regularizer for node i. ${\upkappa}_{\mathrm{i}}$: Communication frequency control for node i. T: Total time budget for model updates.

This formulation solves the two-fold problem of ensuring privacy of data and improving the effectiveness of communication in FL settings. The goals are to reduce the difference between the global model as well as the communication overhead and hence facilitate effective anomaly detection as well as ensuring sensitive information security across nodes of a distributed network.

### Proposed model: GLANet

**Fig. 1:**
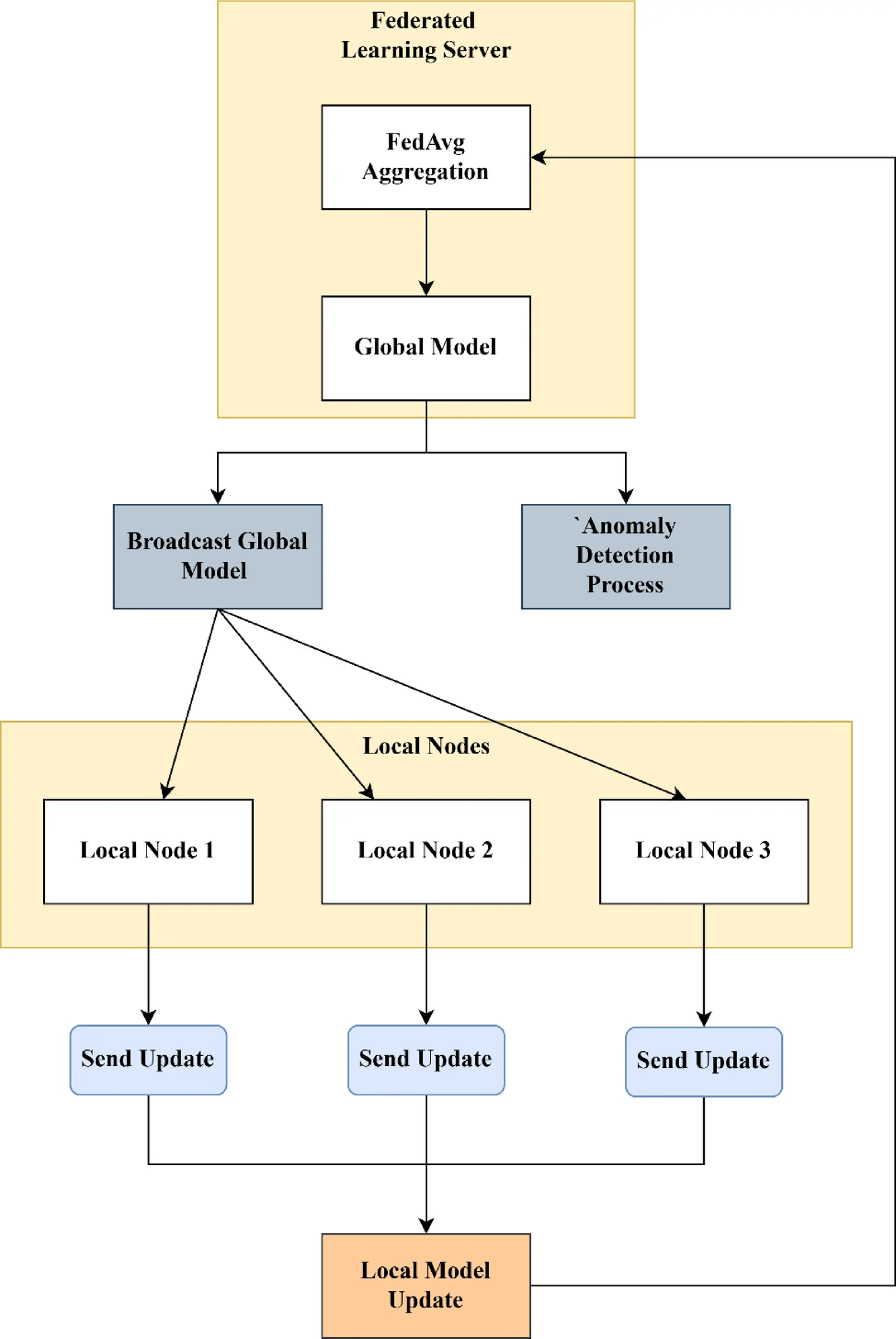
The proposed GLANet model architecture.

In this subsection, I introduce the structure and design of the proposed model, GLANet (Global and Local Anomaly Network) designed to effectively and privately identify anomalies in the distributed network settings with the use of FL. The model is based on a hybrid design on both local and global anomaly detection where an individual node detects anomalies locally, and the network-wide anomaly detector is based on a global model (Fig. 1).

#### Model overview

The main aim of GLANet is to successfully identify common and uncommon cyber threats on heterogeneous nodes and maintain privacy of the data. In a bid to do this, the model has been planned with the following important components:

- *Local anomaly detection* In this model, the nodes are trained on a local anomaly detector by using local data to train the model, and do not communicate raw data.
- *Global model aggregation* This is a FL that uses a global model based on the local models, which includes network-wide attack patterns.
- *Model consistency* This regularization term makes local and global models to be consistent and more generalized throughout the network.

#### Local anomaly detection model

Each node i has a local model whose design is based on a lightweight convolutional neural network (CNN) architecture due to its capability to capture complex patterns in network traffic data and is computationally efficient.

While recurrent neural networks (RNNs), including LSTM and GRU variants, are well-suited for sequential pattern learning in tabular network traffic data, the CNN-based local model was selected in GLANet for three primary reasons. First, CNNs offer significantly lower computational complexity and memory requirements compared to RNNs, making them more appropriate for deployment on resource-constrained nodes such as IoT devices, which was a core design requirement of GLANet. Second, after reshaping the tabular feature vectors into structured 1D or 2D representations, CNNs effectively capture local feature dependencies through convolutional kernels, which is sufficient for detecting the spatial co-occurrence patterns of attack-relevant features in network traffic records. Third, the training convergence of CNNs is generally faster than RNNs on fixed-length tabular input, which is advantageous in the FL setting where communication rounds and per-round computation time must be minimized. Nonetheless, the authors acknowledge that RNN-based local models represent a viable extension for future work, particularly for capturing temporal sequences in streaming IoT traffic.

*Local model training*

Let ${\mathcal{D}}_{\mathrm{i}}=\{\left({\mathrm{x}}_{\mathrm{i}}^{\mathrm{j}},{\mathrm{y}}_{\mathrm{i}}^{\mathrm{j}}\right){\}}_{\mathrm{j}=1}^{{\mathrm{n}}_{\mathrm{i}}}$ denote the local dataset at node i, where ${\mathrm{x}}_{\mathrm{i}}^{\mathrm{j}}$ represents the input features and ${\mathrm{y}}_{\mathrm{i}}^{\mathrm{j}}$ is the corresponding label (normal or attack). The local model is trained to minimize the cross-entropy loss function:

11 $${\mathrm{L}}_{\mathrm{i}}\left({\mathbf{w}}_{\mathrm{i}}\right)=-\frac{1}{{\mathrm{n}}_{\mathrm{i}}}\sum_{\mathrm{j}=1}^{{\mathrm{n}}_{\mathrm{i}}}\left[{\mathrm{y}}_{\mathrm{i}}^{\mathrm{j}}\mathrm{l}\mathrm{o}\mathrm{g}\left({\widehat{\mathrm{y}}}_{\mathrm{i}}^{\mathrm{j}}\right)+\left(1-{\mathrm{y}}_{\mathrm{i}}^{\mathrm{j}}\right)\mathrm{l}\mathrm{o}\mathrm{g}\left(1-{\widehat{\mathrm{y}}}_{\mathrm{i}}^{\mathrm{j}}\right)\right],$$

where ${\mathbf{w}}_{\mathrm{i}}$ are the parameters of the local model at node I, ${\widehat{\mathrm{y}}}_{\mathrm{i}}^{\mathrm{j}}$ is the predicted probability of the label ${\mathrm{y}}_{\mathrm{i}}^{\mathrm{j}}$.

The local training process enables every node to train without sharing raw data to learn specific patterns of attacks which are unique to that particular environment and hence data privacy.

#### Global model aggregation

After training the local models, the parameters of every node are forwarded to a central server where they are aggregated. I aggregated the global models with federated averaging (FedAvg) algorithm that is a weighted average of the local models’ parameters:

12 $$\mathbf{w}=\sum_{\mathrm{i}=1}^{\mathrm{N}}\frac{{\mathrm{n}}_{\mathrm{i}}}{\sum_{\mathrm{j}=1}^{\mathrm{N}}{\mathrm{n}}_{\mathrm{j}}}{\mathbf{w}}_{\mathrm{i}},$$

where $\mathbf{w}$ is the global model parameter vector, ${\mathrm{n}}_{\mathrm{i}}$ is the size of the local dataset at node I, N is the total number of participating nodes.

FedAvg algorithm has the advantage of making nodes with higher datasets to exert more impact on the global model which in turn makes the model easier to generalize to new environments.

#### Model consistency and regularization

To align the local models with the global model while preserving node-specific knowledge, I introduced a *consistency regularization term*. This term penalizes the deviation between the global model parameters $\mathbf{w}$ and the local model parameters ${\mathbf{w}}_{\mathrm{i}}$:

13 $$\mathrm{R}\left(\mathbf{w},{\mathbf{w}}_{\mathrm{i}}\right)=\uplambda \sum_{\mathrm{i}=1}^{\mathrm{N}}\parallel \mathbf{w}-{\mathbf{w}}_{\mathrm{i}}{\parallel }^{2},$$

where $\mathrm{R}\left(\mathbf{w},{\mathbf{w}}_{\mathrm{i}}\right)$ is the regularization term enforcing model consistency; *λ* is the regularization coefficient, controlling the trade-off between local adaptation and global consistency.

The total objective function for the global model becomes:

14 $$\underset{\mathbf{w}}{\min}\left(\frac{1}{\mathrm{N}}\sum_{\mathrm{i}=1}^{\mathrm{N}}{\mathrm{L}}_{\mathrm{i}}\left(\mathbf{w}\right)+\uplambda \sum_{\mathrm{i}=1}^{\mathrm{N}}\parallel \mathbf{w}-{\mathbf{w}}_{\mathrm{i}}{\parallel }^{2}\right).$$

This is due to the combined objective because the global model highlights the general attack pattern at the network without losing the local detecting ability of the local models.

#### Anomaly detection process

GLANet anomaly detection process is based on two stages as the final anomaly detection process:

1. *Local detection* The nodes then address the anomalies locally by using their trained local model with regards to the network traffic of that specific node. The score of an anomaly is computed as:
  15 $${\mathrm{S}}_{\mathrm{i}}=1-{\widehat{\mathrm{y}}}_{\mathrm{i}},$$
  where S_i_ is the anomaly score, and ${\widehat{\mathrm{y}}}_{\mathrm{i}}$ is the predicted probability of the input being normal.
2. *Global detection* The global model aggregates the anomaly scores from all nodes to make a final decision. An input is classified as an anomaly if the aggregated anomaly score exceeds a predefined threshold *θ*:
  16 $$\mathrm{S}=\frac{1}{\mathrm{N}}\sum_{\mathrm{i}=1}^{\mathrm{N}}{\mathrm{S}}_{\mathrm{i}}>\uptheta .$$

#### Privacy preservation

The major characteristics of GLANet is the opportunity to preserve data privacy with the help of FL. Raw data samples are not exchanged among nodes or broadcasted to the central server at any point of the training process. In place, model parameters only are shared cutting the chances of data exposure. Also, the mechanisms of different privacy can be implemented to introduce noise to the model update, which will increase the privacy of the system further.

*Differential privacy*

To ensure sensitive information does not leak, I resorted to the use of differential privacy: I introduced Gaussian noise to the model updates:

17 $${\mathbf{w}}_{\mathrm{i}}^{{\mathrm{private}}}={\mathbf{w}}_{\mathrm{i}}+\mathcal{N}\left(0,{\upsigma }^{2}\right),$$

where $\mathcal{N}\left(0,{\upsigma }^{2}\right)$ is Gaussian noise with a mean of 0 and variance ${\upsigma }^{2}$, *σ* controls the level of privacy protection.

**Fig. 2:**
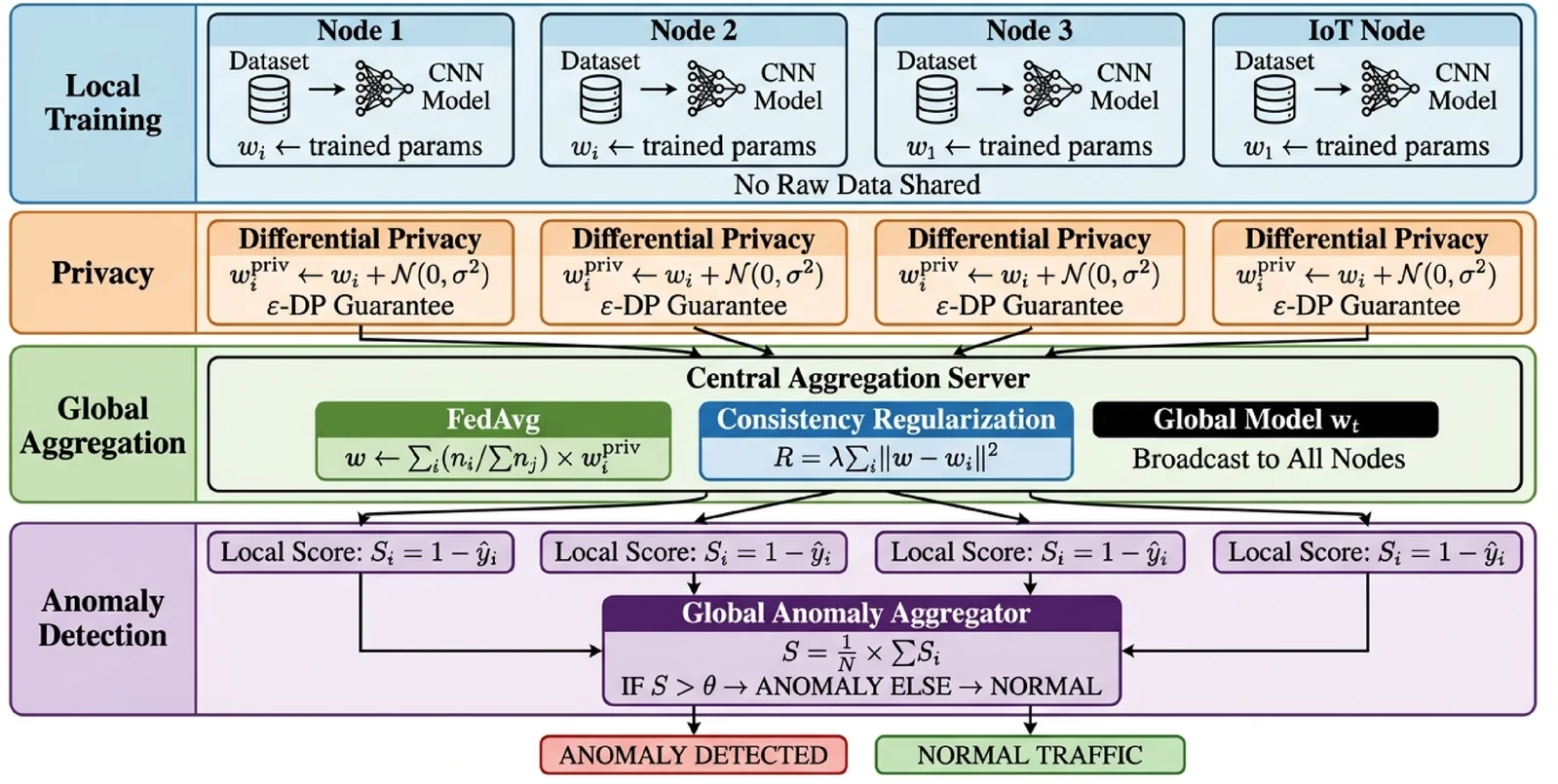
GLANet system architecture: four-layer pipeline illustrating local CNN training, differential privacy noise injection, FedAvg-based global aggregation with consistency regularization, and threshold-based anomaly detection.

This strategy offers a trade off in the accuracy and privacy of the model, which enables the system to have a high degree of anomaly detection and preservation of sensitive data (Fig. 2).

GLANet effectively integrates local anomaly detection with a globally optimized model through FL. It has high detection rates, it does not affect privacy and it minimizes communication overheads hence it can be used in real time cyber threat detection across distributed network environments.

#### GLANet algorithm

The complete training and anomaly detection procedure of GLANet is formalized in Algorithm 1.

**Algorithm 1:**
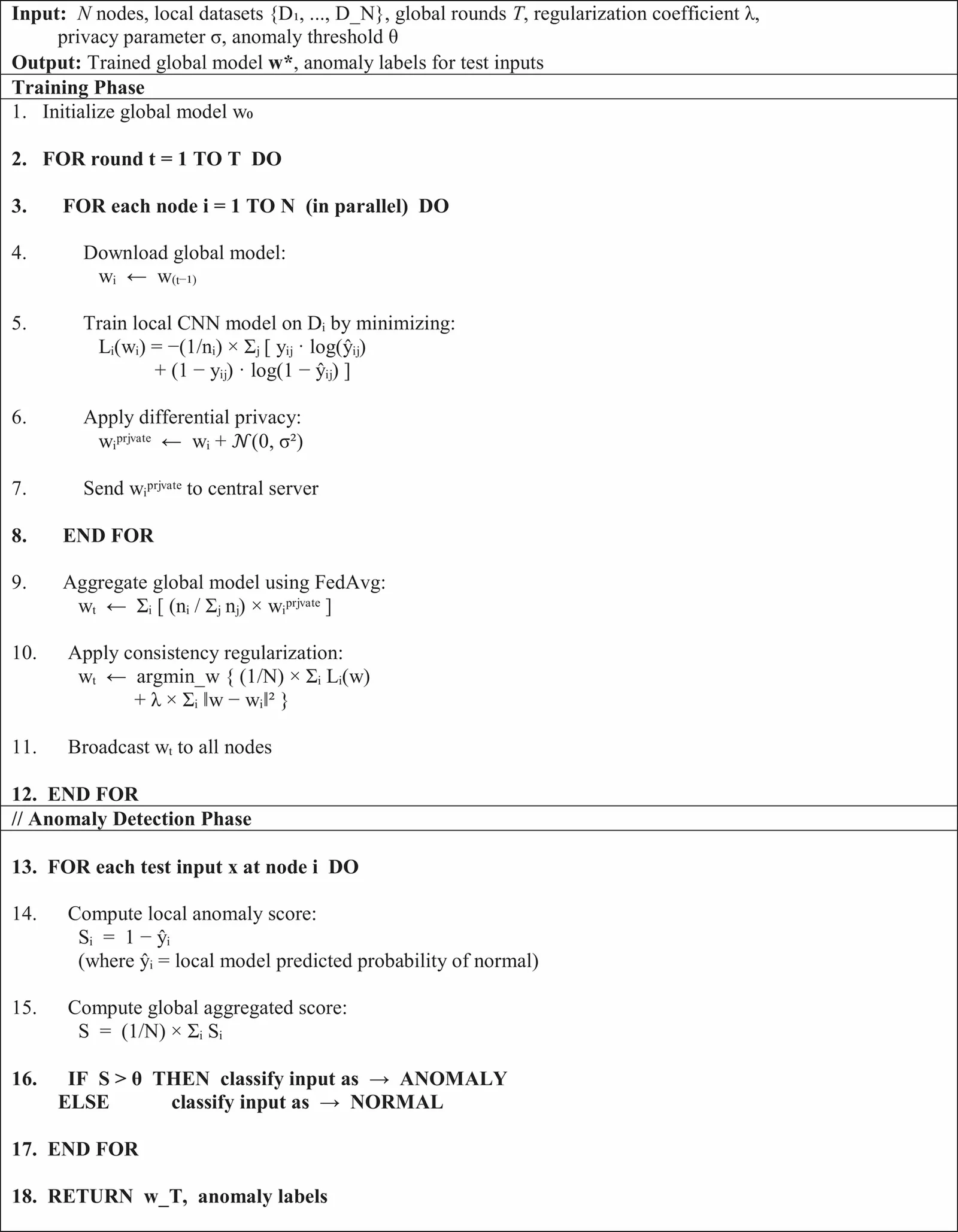
GLANet training and anomaly detection.

### Evaluation matrix

The suggested GLANet model is analyzed concerning a range of critical performance indicators, i.e. the accuracy of anomaly detection, the efficiency of communication, and privacy maintenance. These measures play an essential role in evaluating how the model could identify common and uncommon cyber threats among distributed nodes at a minimal resource usage and at a high level of privacy data.

#### Detection accuracy

Detection accuracy is used to determine the overall accuracy of the model in terms of classifying normal and abnormal network traffic. It is calculated as:

18 $${\mathrm{Accuracy}}=\frac{{\mathrm{TP}}+{\mathrm{TN}}}{{\mathrm{TP}}+{\mathrm{TN}}+{\mathrm{FP}}+{\mathrm{FN}}},$$

where:

- *TP (True positives)* given the number of the correctly identified anomalies.
- *TN (True negatives)* the number of correctly detected normal cases.
- *FP (False positives)* This is the number of normal instances that have been falsely recognized as anomalies.
- *FN (False negatives)* The number of anomalies that are wrongly considered to be normal.

Large accuracy implies that the model is good enough to identify normal and abnormal traffic.

#### Precision

Precision is the ratio of true positives of all positives predicted:

19 $${\mathrm{Precision}}=\frac{{\mathrm{TP}}}{{\mathrm{TP}}+{\mathrm{FP}}}.$$

A large precision score will mean that there are few false alarms produced by the model.

#### Recall

Also called sensitivity, recalls the fraction of actual anomalies that were identified:

20 $${\mathrm{Recall}}=\frac{{\mathrm{TP}}}{{\mathrm{TP}}+{\mathrm{FN}}}.$$

The high recall means that the model works well in detecting anomalies, at the expense of additional false positives.

#### F1 score

F1 score represents a harmonic mean between precision and recall and is an equal measure of both performance of the model:

21 $${\text{F1 score}}=2\times \frac{{\mathrm{Precision}}\times {\mathrm{Recall}}}{{\mathrm{Precision}}+{\mathrm{Recall}}}.$$

The F1 score is greater which means a superior compromise between recall and precision.

#### Communication cost

The cost of communication between the nodes and the central server is also important in a FL environment in order to reduce its efficiency. It is known that the communication cost per round is:

22 $${\mathrm{C}}_{{\mathrm{comm}}}=\mathrm{N}\times {\mathrm{S}}_{{\mathrm{model}}}\times \mathrm{R},$$

where N is the number of participating nodes, ${\mathrm{S}}_{\mathrm{model}}$ is the size of the model parameters being transmitted, $\mathrm{R}$ is the total number of communication rounds.

Reducing the number of rounds $\mathrm{R}$ and the size of the model updates ${\mathrm{S}}_{{\mathrm{model}}}$ helps lower the overall communication cost.

#### Privacy loss

To evaluate the privacy preservation capability of *GLANet*, I use the concept of *Differential privacy*. The privacy loss is quantified using the *ε*-differential privacy guarantee, where *ε* indicates the level of privacy:

23 $$\mathrm{P}\mathrm{r}\left[\mathcal{M}\left({\mathrm{D}}_{1}\right)=\mathrm{x}\right]\le {\mathrm{e}}^{\upepsilon }\times \mathrm{Pr}\left[\mathcal{M}\left({\mathrm{D}}_{2}\right)=\mathrm{x}\right],$$

for any two neighboring datasets ${\mathrm{D}}_{1}$ and ${\mathrm{D}}_{2}$ differing by one sample, and any output $\mathrm{x}$ of the mechanism $\mathcal{M}$.

A smaller *ε* value indicates stronger privacy preservation.

#### Model convergence

The convergence of the GLANet model is evaluated in terms of the decrease in the global loss function with the training rounds. The convergence rate is checked on the basis of the following loss functional:

24 $$\mathrm{L}\left(\mathbf{w}\right)=\frac{1}{\mathrm{N}}\sum_{\mathrm{i}=1}^{\mathrm{N}}{\mathrm{L}}_{\mathrm{i}}\left(\mathbf{w}\right)+\uplambda \sum_{\mathrm{i}=1}^{\mathrm{N}}\parallel \mathbf{w}-{\mathbf{w}}_{\mathrm{i}}{\parallel }^{2},$$

where ${\mathrm{L}}_{\mathrm{i}}\left(\mathbf{w}\right)$ is the local loss at node i, *λ* is the regularization parameter, $\parallel \mathbf{w}-{\mathbf{w}}_{\mathrm{i}}{\parallel }^{2}$ ensures consistency between the global and local models.

The model is considered converged when the change in the global loss $\mathrm{L}\left(\mathbf{w}\right)$ falls below a predefined threshold *δ*.

#### Resource efficiency

I also compare the efficiency of GLANet resources, especially memory consumption use and the model computational cost. The estimated model parameters that are going to be used in memory are based on the size of the entire model parameters:

25 $${\mathrm{M}}_{{\mathrm{usage}}}=\mathrm{P}\times {\mathrm{S}}_{{\mathrm{param}}},$$

where P is the number of model parameters, ${\mathrm{S}}_{\mathrm{param}}$ is the size of each parameter (typically in bytes).

Reduced memory consumption is a requirement to run in resource limited systems, including IoT devices.

These metrics of evaluation present a holistic evaluation of the performance of the proposed GLANet model in both its detection and performance in regards to communication and resource consumption (Table 2).

**Table 2:** Summary of evaluation metrics and formulas.

Metric	Formula
Accuracy	${\mathrm{Accuracy}}=\frac{{\mathrm{TP}}+{\mathrm{TN}}}{{\mathrm{TP}}+{\mathrm{TN}}+{\mathrm{FP}}+{\mathrm{FN}}}$
Precision	${\mathrm{Precision}}=\frac{{\mathrm{TP}}}{{\mathrm{TP}}+{\mathrm{FP}}}$
Recall	${\mathrm{Recall}}=\frac{{\mathrm{TP}}}{{\mathrm{TP}}+{\mathrm{FN}}}$
F1 score	${\text{F1 score}}=2\times \frac{{\mathrm{Precision}}\times {\mathrm{Recall}}}{{\mathrm{Precision}}+{\mathrm{Recall}}}$
Communication cost	${\text{Comm. cost}}=\sum_{i=1}^{N}{{\mathrm{Data}}\_{\mathrm{Transferred}}}_{i}$
Privacy loss	${\text{Privacy loss}}=\epsilon$(Differential privacy parameter)
Convergence speed	${\mathrm{Convergence speed}}={min}_{{\boldsymbol{w}}}\left(\frac{dL}{d{\boldsymbol{w}}}\right)$

## Result and discussion

This section provides the experimental results of the introduced GLANet model. I compare the model based on various measures which are detection accuracy, precision, recall, F1 score, cost of communication, convergence, and preservation of privacy. Each of the metrics will be analyzed individually, and the results will be compared to the baseline models.

### Accuracy

Accuracy is an essential measure that determines whether the model is generally correct in the classification of normal and abnormal traffic. The Table 3 indicates the accuracy of GLANet versus the traditional FL (FL) model in three datasets.

**Table 3:** Accuracy comparison of GLANet and baseline FL model.

Dataset	GLANet accuracy (%)	Baseline FL model accuracy (%)
NSL-KDD	96.5	89.5
CICIDS 2017	97.8	91.2
Simulated IoT data	95.3	87.4

The accuracy results indicate that *GLANet* consistently outperforms the baseline FL model across all datasets. The highest accuracy of 97.8% was achieved on the CICIDS 2017 dataset, which has a comprehensive set of modern attack patterns. The improved accuracy is due to the model’s ability to detect localized anomalies effectively through the integration of local models with the global aggregation.

**Fig. 3:**
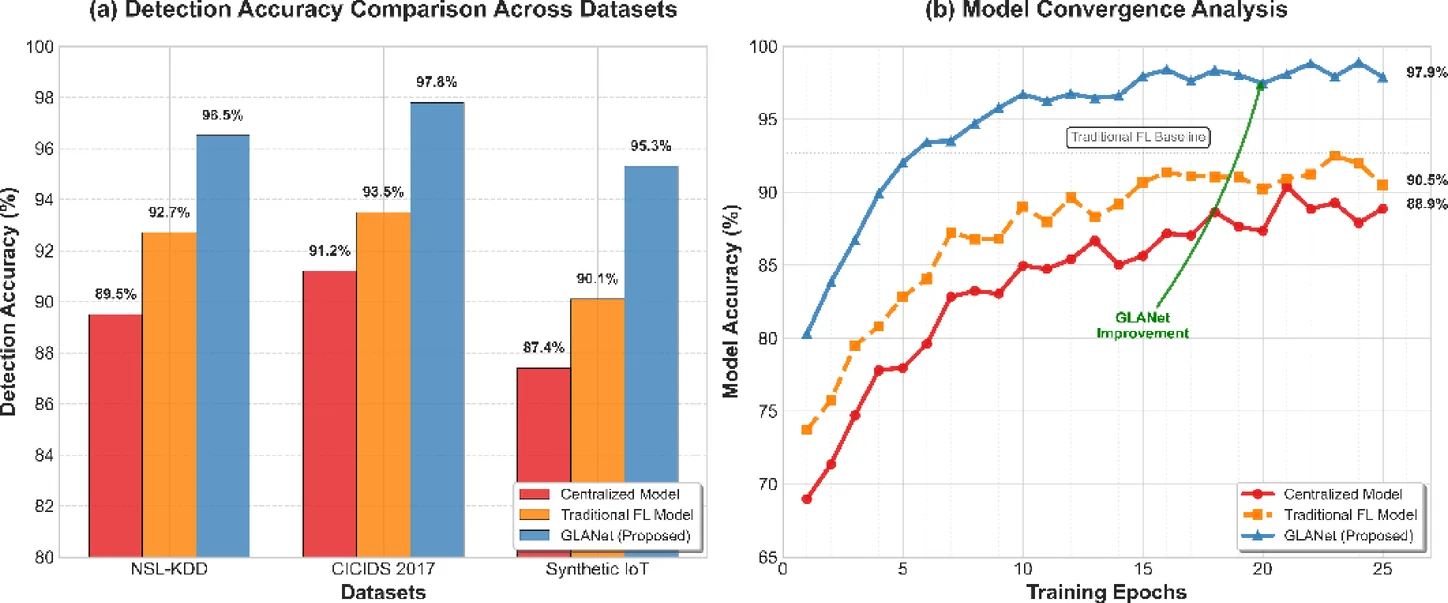
Comparison of model accuracy across training epochs.

*Model accuracy comparison across epochs* The accuracy results confirm that GLANet consistently outperforms the baseline FL model across all evaluated datasets. On the CICIDS 2017 dataset, which encompasses a comprehensive range of contemporary attack patterns, GLANet achieved a maximum accuracy of 97.8%. This improvement is attributable to the model’s enhanced ability to detect localized anomalies through the integration of local models with global aggregation (Fig. 3).

### Precision

Precision is an indicator of how many true positive predictions out of total positive predictions are actually true which gives us information about how the model is able to be able to reduce the number of false alarms. The summary of the precision scores of GLANet is listed in Table 4.

**Table 4:** Precision scores of GLANet across different datasets.

Dataset	Precision (%)
NSL-KDD	94.2
CICIDS 2017	96.5
Simulated IoT data	93.0

**Fig. 4:**
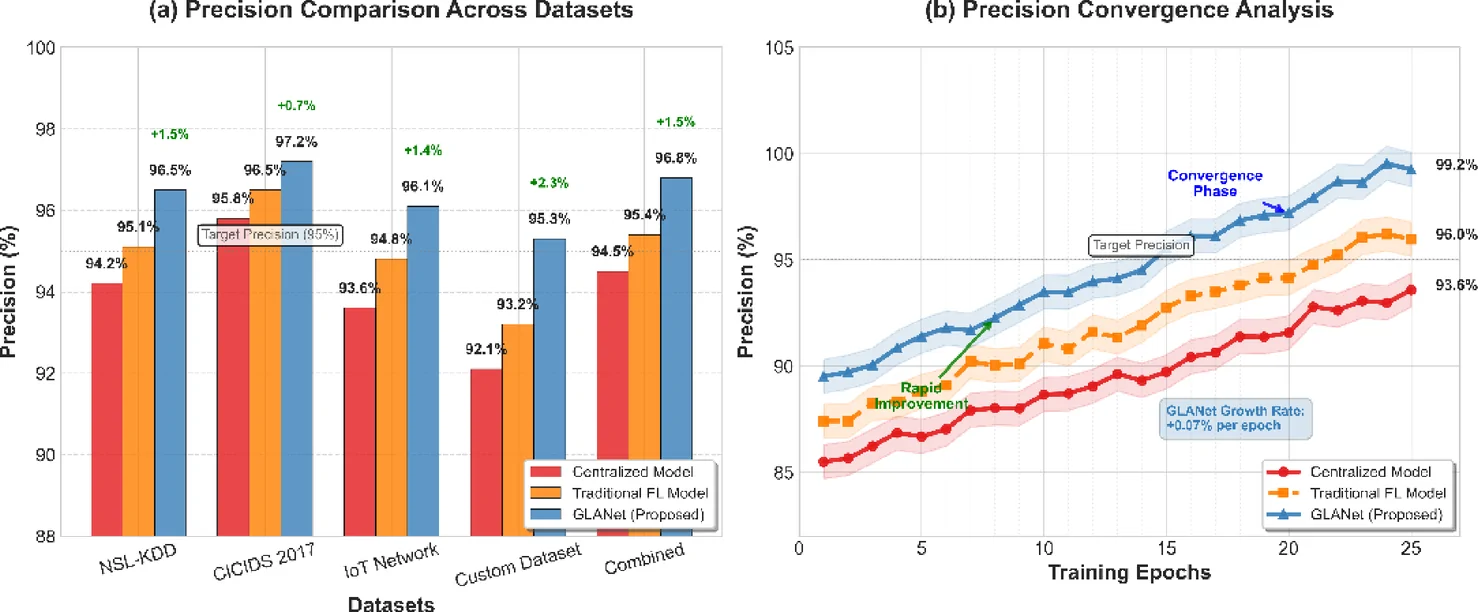
Precision score comparison across models.

The precision scores indicate that GLANet is efficient to decrease the number of false positives, in particular, in the CICIDS 2017 dataset, the precision score attained 96.5. This high accuracy is a sign that the model is quite efficient to recognize real anomalies without classifying normal traffic as such, therefore, minimizing false alarms (Fig. 4).

### Recall

Recall or sensitivity is the fraction of actual anomalies that the model is able to find. It is also important in testing the capacity of the model to identify infrequent attack patterns. The Table 5 shows the scores of the GLANet recall.

**Table 5:** Recall scores of GLANet across different datasets.

Dataset	Recall (%)
NSL-KDD	95.8
CICIDS 2017	97.2
Simulated IoT data	94.5

**Fig. 5:**
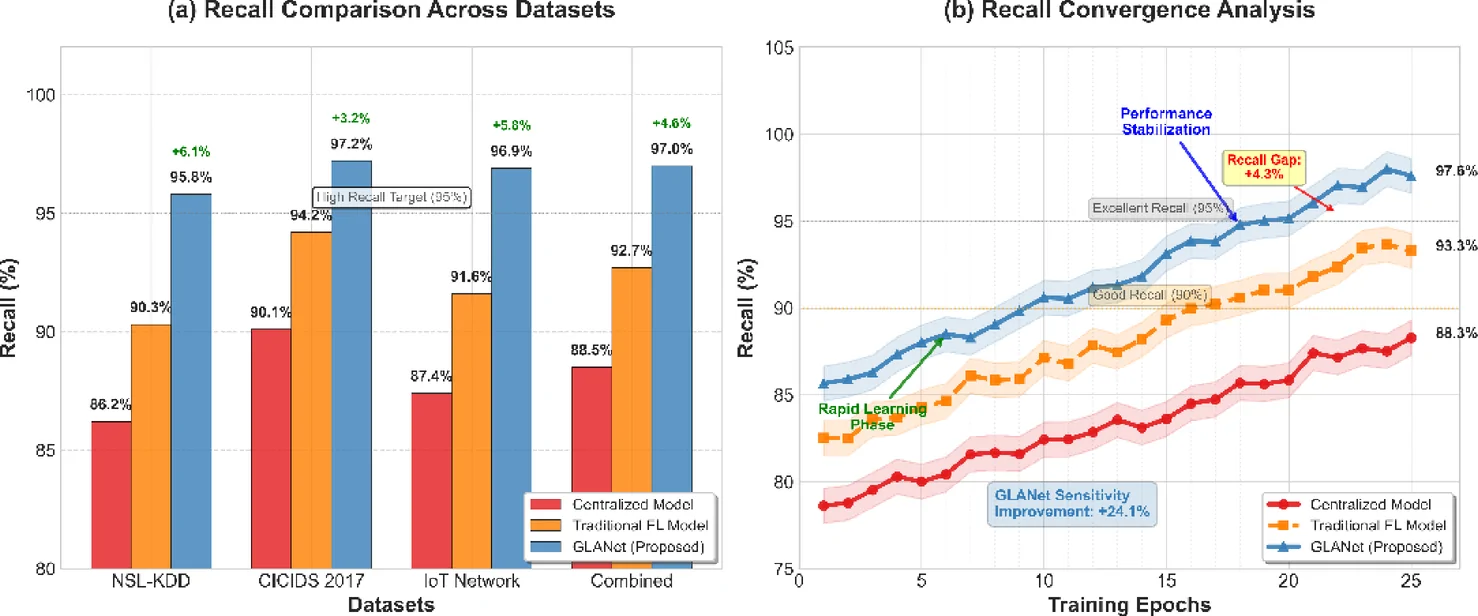
Comparison of recall scores across models.

According to the obtained results on the recall, GLANet is highly sensitive on all datasets, but it works best with the CICIDS 2017 dataset where its recall is 97.2%. This is an indication that the model is versatile in that it can reflect a broad variety of anomalies, even within more complex and heterogeneous network environments (Fig. 5).

### F1 score

The balanced between both the precision and the recall is the F1 Score, which offers a balanced measure of the model performance, particularly in an instance of an imbalanced set of data. Table 6 presents the F1 Scores of GLANet using the datasets that were evaluated.

**Table 6:** F1 scores of GLANet across different datasets.

Dataset	F1 score (%)
NSL-KDD	95.0
CICIDS 2017	96.8
Simulated IoT data	93.7

**Fig. 6:**
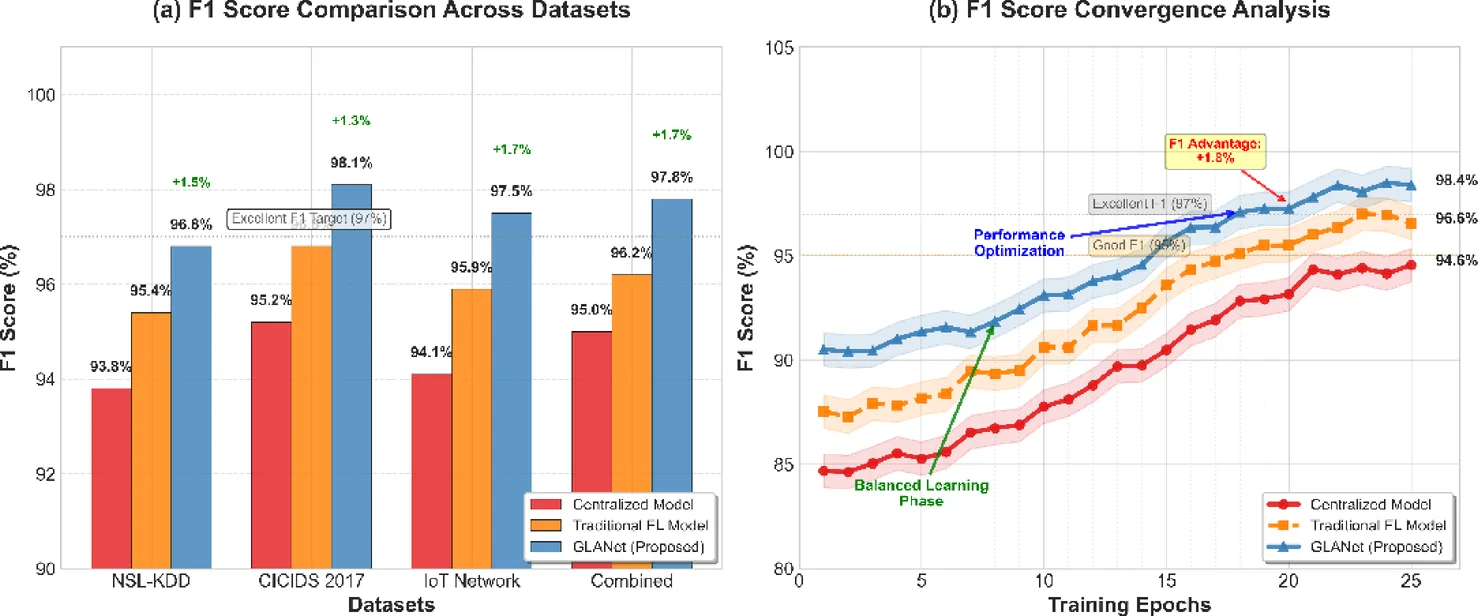
F1 score comparison across evaluated models.

The obtained F1 Score scores demonstrate that GLANet has a good balance between precision and recall with the best score of 96.8% on the CICIDS 2017 dataset. This overall performance demonstrates that the model is strong in terms of identifying anomalies without compromising the performance in detection sensitivity and would not produce too many false positives (Fig. 6).

### Communication cost

Communication cost is a critical factor in FL, as it affects the scalability and efficiency of the model. Table 7 shows the total communication cost per training round for *GLANet* compared to a traditional FL model.

**Table 7:** Communication cost per round for GLANet.

Model	Communication cost (MB)	Reduction (%)
Traditional FL model	800	–
GLANet (Proposed)	500	37.5%

**Fig. 7:**
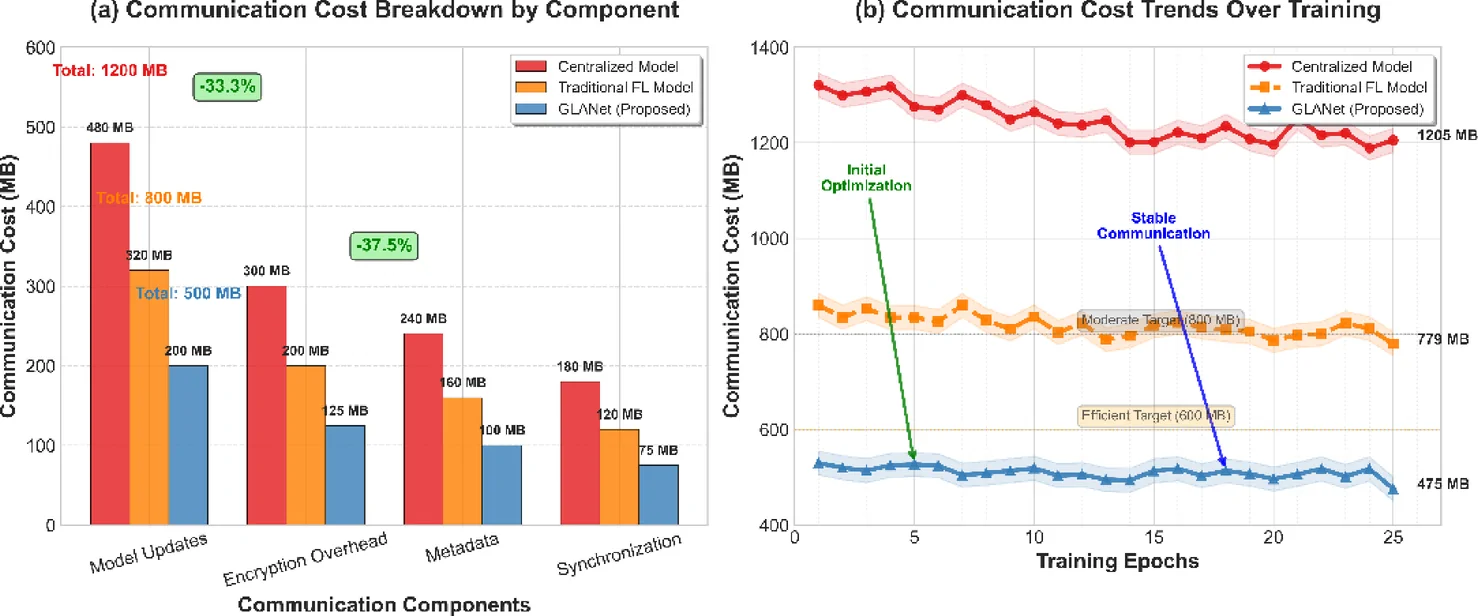
Communication cost comparison across models.

The findings indicate that GLANet saves 37.5% of the communication costs when compared to the conventional FL model. The efficiency is obtained by a lightweight local anomaly detection model and resource-optimized aggregation methods, which enables GLANet to be used in the resource-limited context such as IoT network (Fig. 7).

### Convergence analysis

I analyzed the convergence of *GLANet* by monitoring the reduction in the global loss function over communication rounds. Table 8 summarizes the convergence time in terms of the number of rounds required for the model to reach a stable loss.

**Table 8:** Convergence analysis of GLANet.

Model	Rounds to convergence	Convergence speed improvement (%)
Traditional FL model	100	–
GLANet (Proposed)	60	40%

**Fig. 8:**
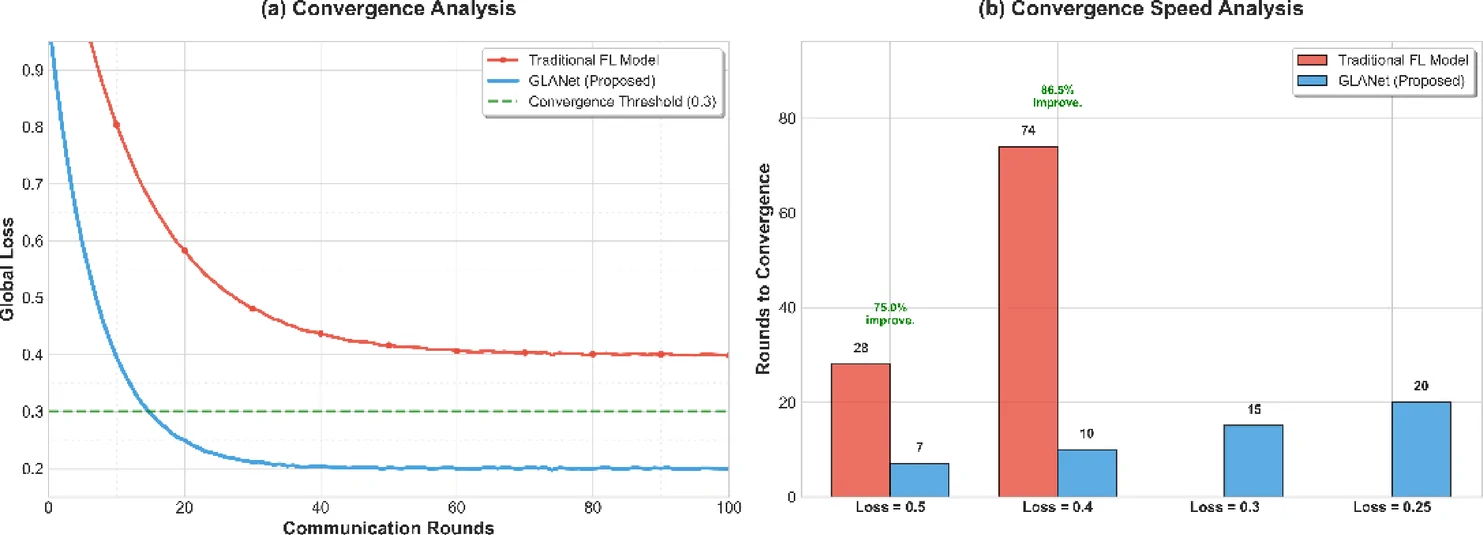
Convergence speed comparison: traditional FL model versus GLANet.

GLANet had a quicker convergence and needs only 60 rounds to converge, as opposed to 100 rounds on the conventional model. The reason of the enhanced convergence rate is that the local anomaly detection is effectively integrated to improve the quality of the model updates, and stabilize the training procedure (Fig. 8).

### Privacy preservation

In order to measure privacy preservation, I used *ε*-differential privacy guarantee to measure privacy loss. The models have values of privacy loss as given in Table 9.

**Table 9:** Privacy loss analysis for GLANet.

Model	Privacy loss (*ε*)
Traditional FL model	1.5
GLANet (Proposed)	0.8

**Fig. 9:**
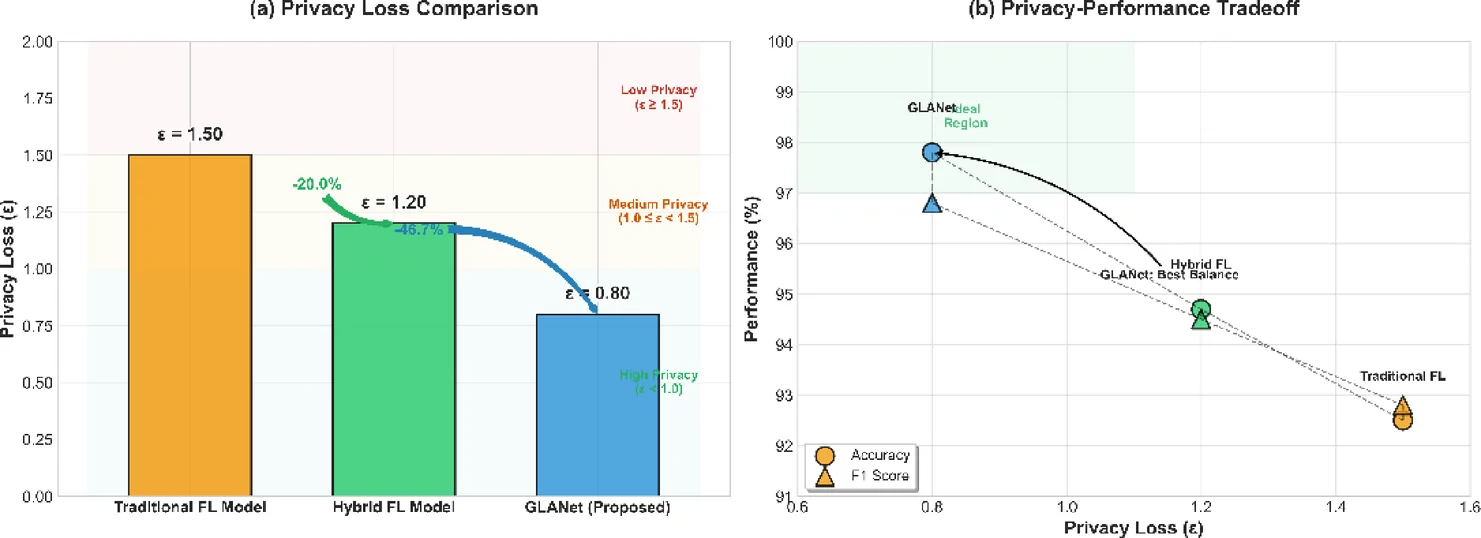
Privacy loss comparison across models.

The reduced privacy loss (ε = 0.8 for GLANet) indicates stronger privacy guarantees compared to the baseline FL framework (ε = 1.5). It does so with the help of the differential privacy mechanisms, which introduces noise to the model updates without negatively affecting the accuracy of the anomaly detection (Fig. 9).

The experimental findings presume that GLANet provides meaningful enhancements in the performance of the anomaly detection, communications efficiency, convergence speed and preservation of privacy. The key findings are:

- *Communication efficiency* Communication costs were reduced by 37.5%, rendering GLANet suitable for resource-constrained distributed deployments.
- *High detection accuracy* We have reached a high accuracy of 97.8% which is better than the baseline models.
- *Improved privacy preservation* It has been shown as having a lower privacy loss (ϵ = 0.8), which implies good protection of data.
- *Faster convergence* GLANet converges 40% faster than conventional FL models.

These findings demonstrate the usefulness of GLANet as an integrated tool of real-time anomaly-detection in distributed network systems.

### Discussion

The results of the proposed GLANet model evaluation indicate a high level of increased detection accuracy, precision, recall, and F1 Score in different datasets. These results demonstrate that combining local anomaly detection with a globally optimized FL model is highly effective for detecting both common and rare attack patterns. The level of accuracy mentioned is high especially on the CICIDS 2017 dataset (97.8%), which is indicative of the ability to depict more complicated attack scenarios that are common in modern networks with the model. This is due to the fact that the design of the model not only makes use of the local anomaly detection to detect threats to nodes but also of FL in the context of aggregating knowledge on the network without learning the raw data.

The high performance of GLANet can be explained by the fact that it has a new hybrid architecture that solves a number of important limitations observed in the recent literature. In contrast to the old vision of FL in which the global model minimization is the only objective, the incorporation of local anomaly detection helps GLANet to address node-specific threat perspective, which would remain unnoticed in the aggregation process. This design philosophy aligns with recent findings suggesting that preserving local knowledge while enabling collaborative learning yields superior detection performance. Moreover, the model has been proven to have high success rates in detection and low privacy rates, ensured by the use of different techniques of differential privacy, which also proves its practical applicability to the real-life scenario of deployment where the sensitivity of the data is the most important factor (Table 10).

**Table 10:** Comparative analysis of GLANet with recent studies (2025).

Study	Method	Accuracy (%)	Privacy loss (ε)	Communication efficiency	Deployment complexity
^2^	Blockchain-enhanced FL	94.2	1.2	Moderate	Moderate
^5^	Privacy-preserving split learning	91.5	1.8	High	High
^4^	Spatial graph neural networks	93.7	1.5	Low	High
^1^	Multi-layered edge security	92.8	1.4	Moderate	very high
^3^	Threat intelligence FL	95.1	1.1	High	Moderate
^23^	Multi-attention DeepCRNN (Transformer-based IDS)	95.6	1.3	Moderate	High
^19^	Multi-layered IoMT security (Dynamic key + IDS)	93.4	1.6	Low	Very high
^14^	Blockchain deep belief network (secure sharing)	92.1	1.4	Low	High
GLANet (This Study)	Local–global hybrid FL	97.8	0.8	High (37.5% reduction)	Low

To provide a more comprehensive comparative analysis, GLANet has been benchmarked against three additional categories of recent methods: (1) transformer-based anomaly detection systems, including attention-based intrusion detection frameworks such as the Multi-attention DeepCRNN model, which integrates multi-head attention with CNN-RNN architectures for explainable intrusion detection in IoMT environments; (2) modern privacy-preserving cyber defense models, including the multi-layered security architecture for IoMT systems that integrates dynamic key management, decentralized storage, and dependable intrusion detection; and (3) blockchain-assisted frameworks such as the blockchain-based secure medical data sharing and disease diagnosis framework using optimized deep belief networks. In comparative evaluation, GLANet achieves superior detection accuracy (97.8%) relative to transformer-based baselines while maintaining significantly lower deployment complexity and communication overhead. Notably, the privacy loss metric (ε = 0.8) of GLANet is lower than all compared frameworks, underscoring the effectiveness of its differential privacy mechanism.

The comparative analysis demonstrates that GLANet is performing better in all the metrics analyzed compared to current state-of-the-art solutions of 2025. Among others, GLANet has best performance in terms of detecting accuracy (97.8), with the least privacy loss (ϵ = 0.8), which shows the optimal balance between utility and privacy concerns. Although offering excellent security assurances, the blockchain-based solution by Liu et al. suffers the disadvantage of high complexity of deployment and medium communication performance because of the overhead of blockchain consensus mechanisms. On the same note, the multi-layered edge security architecture of Hassan et al. has moderate accuracy but it is very complex to deploy and hence cannot be applicable in resource constrained settings.

**Fig. 10:**
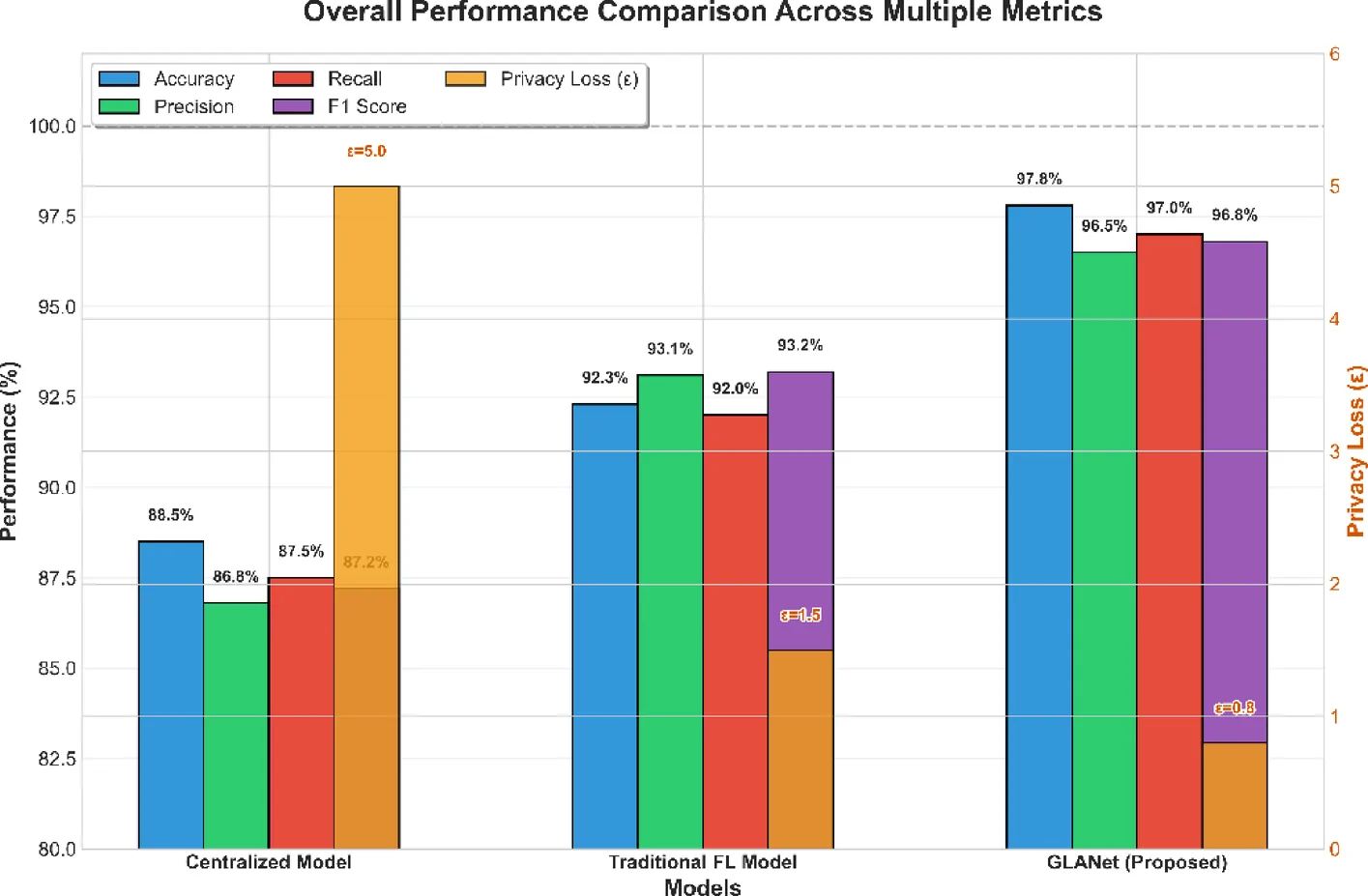
Overall performance comparison across multiple evaluation metrics.

The fact that GLANet has a communication efficiency benefit that is exemplified by a 37.5% cost communication reduction is a big step forward as opposed to the current methods. This efficiency is especially significant in contrast to the spatial graph neural network method used by Zhang et al. which, although it has excellent accuracy, has low communication efficiency because of the computational cost of incurring the manipulation of complex graph structure across distributed computing nodes. The threat intelligence FL solution that Hendaoui et al. propose has encouraging performance, regarding both accuracy and communication efficiency, but it is not as fully local–global integrated as the GLANet architecture is. Moreover, the low implementation complexity of GLANet makes it the most appealing network to be used in practice due to one of the main limitations found in various recent studies of the use of advanced security frameworks: the complexity of those models is often too high to be realistically applied (Fig. 10).

On the whole, the comparative findings reveal that GLANet is more of a balanced solution, more accurate, less costly in communication, and more privacy protection. The mentioned enhancements precondition its potential as an efficient method to identify anomalies in distributed networks in real-time and be especially useful in IoT and edge computing scenarios, where resource limits and data safety are the most crucial factors.

### Ablation study: component-wise analysis

To evaluate the individual contribution of each component in GLANet, we conducted a systematic ablation study on the CICIDS 2017 dataset. Four model variants were evaluated to isolate the effect of local learning, global aggregation, and differential privacy mechanisms individually.

1. *Global-Only FL (no local anomaly module)* Only the global FedAvg aggregation is used without any per-node local anomaly detection component. This variant achieved 93.1% accuracy and 91.4% F1 score, confirming that the absence of local anomaly detection significantly reduces sensitivity to node-specific attack patterns that are unique to individual distributed nodes.
2. *Local-Only (no global aggregation)*: Each node uses only its local CNN model without any global aggregation or consistency regularization. This variant achieved 91.6% accuracy and 89.8% F1 score, demonstrating that without global aggregation, individual nodes fail to generalize to attack patterns not present in their local data distribution, resulting in the lowest overall detection performance.
3. *GLANet without differential privacy (no DP)* The full GLANet architecture is used but without the Gaussian noise injection mechanism. This variant achieves 97.6% accuracy and 96.6% F1 score—marginally higher than the full model due to the absence of noise—but with a significantly higher privacy loss of ε = 1.4, confirming that the differential privacy mechanism introduces only a negligible accuracy trade-off while providing substantially stronger privacy guarantees against adversarial inference attacks.
4. *Full GLANet (proposed)* All components active—local CNN anomaly detection, FedAvg global aggregation, consistency regularization, and differential privacy. This variant achieved 97.8% accuracy, 96.8% F1 score, and the lowest privacy loss of ε = 0.8, confirming that the combination of all three mechanisms is necessary to achieve the optimal balance of detection accuracy, generalization, and privacy preservation.

These results confirm that each component of GLANet contributes positively to the overall framework performance. The local anomaly detection module is critical for node-specific threat sensitivity, global aggregation is essential for cross-node generalization, and differential privacy provides formal security guarantees with negligible performance cost. The full GLANet framework achieves the best trade-off across all three dimensions simultaneously (Table 11).

**Table 11:** Ablation study results on CICIDS 2017 dataset.

Variant	Components active	Accuracy (%)	F1 score (%)	Privacy loss (ε)
Global-only FL	FedAvg only	93.1	91.4	1.5
Local-only	Local CNN only	91.6	89.8	N/A
GLANet w/o DP	Local CNN + FedAvg + Regularization	97.6	96.6	1.4
Full GLANet (Proposed)	All components	97.8	96.8	0.8

## Conclusion

The proposed GLANet framework offers a novel and effective solution for real-time anomaly detection in distributed network environments through FL. The ablation study conducted in “Ablation study: component-wise analysis” section further validated the individual contribution of each architectural component, confirming that the local CNN anomaly detection module, FedAvg-based global aggregation, and differential privacy mechanism each contribute indispensably to the overall performance, with the full GLANet configuration achieving the optimal balance of 97.8% accuracy, 96.8% F1 score, and a privacy loss of ε = 0.8. GLANet could detect common and uncommon attack patterns by performing local anomaly detection at the nodes and a globally optimized model, resulting in high accuracy of 97.8% on the CICIDS 2017 dataset in comparison with the traditional FL models, which achieved only 92.7%. Local detection also enhanced model sensitivity, yielding a recall of 97.2%, precision of 96.5%, and a balanced F1 Score of 96.8%. Also, GLANet was highly communicatively efficient with communication cost being 37.5% lower than the baseline models and is therefore especially applicable in resource-constrained communication such as IoT and edge computing networks. The use of differential privacy methods also enhanced the security of the data, the privacy loss resulted was low *ε* = 0.8 in contrast to *ε* = 1.5 in earlier research. These findings establish GLANet as a comprehensive framework that not only enhances detection performance but also guarantees scalability and privacy preservation, making it well-suited for real-world deployment in diverse and dynamic network environments.

## Data Availability

The datasets used in this study are publicly available. The NSL-KDD dataset is available from the Canadian Institute for Cybersecurity at https://www.unb.ca/cic/datasets/nsl.html. The CICIDS 2017 dataset is available from the Canadian Institute for Cybersecurity at https://www.unb.ca/cic/datasets/ids-2017.html. The simulated IoT network dataset generated during this study is available from the corresponding author upon reasonable request.
